# Avocado (*Persea americana*) pulp improves cardiovascular and autonomic recovery following submaximal running: a crossover, randomized, double-blind and placebo-controlled trial

**DOI:** 10.1038/s41598-020-67577-3

**Published:** 2020-07-01

**Authors:** Fernando H. Sousa, Vitor E. Valenti, Leticia C. Pereira, Rafaela R. Bueno, Sara Prates, Amanda N. Akimoto, Mojtaba Kaviani, David M. Garner, Joice A. T. Amaral, Luiz Carlos de Abreu

**Affiliations:** 1 Department of Morphology and Physiology, University Health Center ABC, Santo Andre, SP Brazil; 2 Autonomic Nervous System Center (CESNA), Sao Paulo State University (UNESP), Av. Hygino Muzzi Filho, 737, Mirante, 17, Marilia, SP 525-900 Brazil; 3 Post-Graduate Program in Physical Therapy, Sao Paulo State University (UNESP), Presidente Prudente, SP Brazil; 4 School of Nutrition and Dietetics, Acadia University, Wolfville, NS Canada; 5 Cardiorespiratory Research Group, Department of Biological and Medical Sciences, Faculty of Health and Life Sciences, Oxford Brookes University, Headington Campus, Gipsy Lane, Oxford, OX3 0BP UK; 6 Department of Pediatric, Faculty of Medicine, University of São Paulo, São Paulo, Brazil

**Keywords:** Neuroscience, Cardiology, Physiology

## Abstract

Previous studies have demonstrated that regular avocado consumption presents advantageous effects on cardiovascular system. However, little attention has been paid to the use of avocado as a dietary supplement, in particular, for individuals involved in physical exercise training. Therefore, this study aims to evaluate the effect of acute avocado pulp intake on cardiovascular and autonomic recovery subsequent to moderate exercise. Using a crossover, randomized, double-blind and placebo-controlled trial design, 16 healthy female adults underwent two protocols: Avocado pulp (600 mg in capsule) and placebo (600 mg starch in capsule). After the ingestion of Avocado pulp or placebo, the subjects were seated for 60 min at rest, followed by running on a treadmill at a submaximal level and then remained seated for 60 min during recovery from the exercise. Heart rate (HR), heart rate variability (HRV) [rMSSD, SD1, HF (ms^2^)] and skin conductance were evaluated before and during exercise, as well as during recovery. HR, systolic blood pressure, HRV and skin conductance recovered faster when subjects were given avocado pulp prior to exercise. In conclusion, avocado pulp improved cardiovascular and autonomic recovery after exercise, suggesting a reduced risk of cardiovascular events after exertion. The current results support the beneficial effects of ingestion of avocado prior to submaximal treadmill running.

## Introduction

The autonomic nervous system is controlled by central areas in the brain and brainstem^1,2^. During exercise the autonomic nervous system is responsible for physiological changes required to meet the contending demands of skin blood flow (thermoregulation) and the functioning muscles (metabolic loads), to maintain blood pressure and optimal organ perfusion^1^. This is key to keeping the body healthy under stressful conditions^1,2^. In the first few seconds of exercise, reduction of parasympathetic activity increases heart rate (HR), followed by an elevation in sympathetic activity, inducing an increase in arterial blood pressure, cardiac output and blood flow in skeletal muscles. Immediately after exercise, parasympathetic reentrance and weakened sympathetic activity occur as characteristics of autonomic recovery^3^.

The period of autonomic and cardiovascular recovery after aerobic exercise is an appropriate time to evaluate the risk of cardiovascular events, including impaired heart rhythm and abnormal or irregular heartbeats^3–6^. HR variability (HRV) has proven useful in examining autonomic recovery after exercise^7,8^. HRV is a simple, reliable and non-invasive method of evaluating inter beat interval (IBI) fluctuations with the intention of detecting changes of the autonomic HR control^9^.

These conditions during exercise training has been studied in animals and humans owing to its numerous beneficial effects on the metabolic and cardiovascular systems^10^. Thus, acute interventions to speed up cardiovascular and autonomic recovery after exercise is a key issue to be investigated, as it may decrease the risk of cardiovascular events during and after significant effort^3,4^.

Cardiac disorders are treated with pharmacotherapies under medical supervision^11,12^ and could be an advisable technique to be implemented prior to exercise. Nevertheless, clinical side-effects of pharmacotherapies are a significant hazard^13^.

In an attempt to avoid medical and physiological problems due to pharmacological side effects during exercise, the study of natural remedies is an appropriate area to explore for complementary therapies in patients who take part in aerobic exercise training^14^. A promising foodstuff that has received attention is the Avocado (*Persea americana*). Phenolic acids and flavonoids have both been found in avocado seeds^15^, suggesting a supportive effect on cardiovascular health^16^.

Epidemiological studies reported that chronic avocado oil ingestion reduced triglycerides, C-reactive protein, LDL and VLDL cholesterol in a rat model of sucrose-induced metabolic changes^17^ and, that chronic avocado pulp treatment improves anti-platelet and anti-thrombotic activities^18^. Of late, a randomized controlled trial demonstrated acute beneficial properties of polyunsaturated and monounsaturated fatty acid from avocado on glycemic and vascular markers during an acute postprandial challenge in middle-aged, overweight or obese subjects^19^. Furthermore, previous studies have reported that chronic ingestion of aqueous avocado leaf extract is beneficial in improving cardiovascular parameters in rats^20^ and the inclusion of one fresh avocado per day in a moderately fatty diet supports an improvement in cholesterol levels^21^.

The above-mentioned studies promote the hypothesis that avocado pulp could present helpful effects similar to avocado fruit, avocado oil and avocado extracts. Yet, it is uncertain in the scientific literature whether avocado pulp has an advantageous influence on cardiovascular and autonomic parameters. In light of the above deliberations, the following questions were highlighted: Is avocado pulp able to improve cardiovascular recovery after exercise? In an optimistic case, is it owing to positive influences on the autonomic nervous system? In order to answer these questions, this study was commenced to evaluate the acute effects of avocado pulp on autonomic and cardiovascular recovery after aerobic exercise.

## Method

### CONSORT statement

This study is in agreement with the CONSORT (Consolidated Standards of Reporting Trials) statement. The current investigation includes details of the study population and settings; subject selection (inclusion or exclusion criteria); methods of randomization and blinding; efficacy, and safety procedures. The study design and statistical procedures have been described. The details were provided concerning trial design, participants, interventions, outcomes, sample size, randomization, blinding and statistical methods^22^.

### Trial design

This is a crossover, randomized, double-blind and placebo-controlled trial. The project was registered with the Brazilian Registry of Clinical Trials, which is accredited by the World Health Organization (https://www.paho.org/hq/index.php?option=com_content&view=article&id=5488:2011-international-clinical-trial-registry-platform-ictrp&Itemid=820&lang=en) (https://www.ensaiosclinicos.gov.br/rg/RBR-9cvrrs/Protocol number: RBR-9cvrrs) on 22/01/2019. The study was completed at the Autonomic Nervous System Center, Sao Paulo, State University, UNESP, Marilia, SP, Brazil.

### Subjects

**Figure 1:**
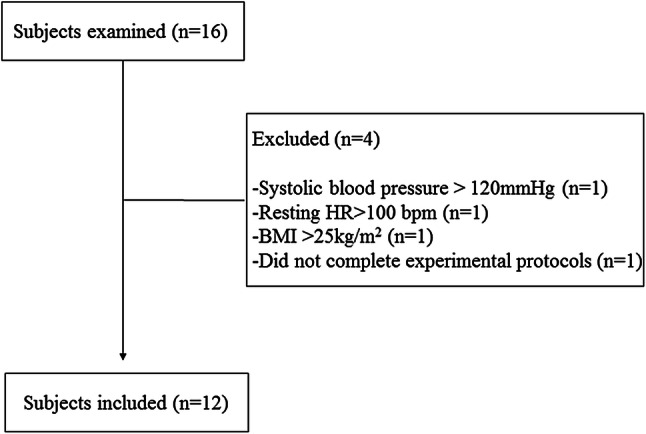
Flowchart.

Initially, 16 healthy, physically active, college educated, females were assessed. Three subjects were excluded from the study because of higher resting SBP > 120 mmHg, resting HR > 100 bmp, BMI > 25 kg/m^2^ or < 18.5 kg/m^2^. Also, one subject was unable to complete all the required stages of the experimental protocol. Therefore, a total of twelve subjects completed the study (Fig. 1).

Subjects were excluded under the following conditions: cardiorespiratory, neurological, musculoskeletal, renal, metabolic, endocrine and other reported disorders that prohibited the successful completion of the protocols, those subjects using medications, smoking, and those had a resting root mean square of successive differences (RMSSD) index < 15 ms^9^. Also, subjects participated on 10th and 15th days and between the 20th and 25th days of the menstrual cycle, in order to avoid influence of the luteal and follicular phase, respectively^23^. Sedentary, inadequately active or overactive subjects evaluated according to the International Physical Activity Questionnaire (IPAQ)^24^ explained and validated in Brazilian Portuguese^25^ were excluded.

This study was accomplished at the Autonomic Nervous System Center, Sao Paulo State University, UNESP, Marilia, SP, Brazil.

### Initial assessment

The initial screening was completed to obtain classification information of the individuals and their eligibility criteria. An anamnesis was performed to confirm the absence of reported disorders, the use of pharmacotherapies, to measure cardiovascular variables, to complete a questionnaire on the level of physical activity and to assess the appropriateness of participating in the experimental protocol.

The subjects’ characteristics were documented by collecting data such as age, mass, height, HR, respiratory rate (RR), SBP, diastolic blood pressure (DBP) and BMI. Levels of physical activity for the subjects was evaluated by the enforcing the IPAQ^24,25^ questionnaire. Consistent with their classifications, individuals were deemed sedentary, inadequately active, active or very active.

The anthropometric measurements were completed according to the format described by Lohman et al.^26^. Stature was assessed by a stadiometer (ES2020, Sanny, Brazil) with an accuracy of 0.1 cm. The mass was attained via a digital scale (W200/5, Welmy, Brazil) with an accuracy of 0.1 kg. Fat percentage was calculated via tetrapolar bioimpedance analysis (Omron, Sao Paulo, SP, Brazil).

### Outcomes

#### Cardiorespiratory variables

During the SBP and DBP measurements, the subjects remained seated. SBP and DBP were obtained indirectly by auscultation with a stethoscope (Premium, Barueri, SP, Brazil) and a calibrated aneroid sphygmomanometer (Premium, Barueri, SP, Brazil) on the subjects’ left arm. Respiratory rate (RR) was considered by counting the respiratory cycles during one minute while the subject was unaware, with the aim of avoiding potential influences and changes in the subjects’ measured respiratory patterns.

For consistency, the same investigator performed the SBP and DBP measurements. The HR was assessed via the Polar RS800cx HR monitor (Polar Electro, Finland).

#### HRV analysis

HRV analysis was accomplished according to the instructions from the Task Force of the European Society of Cardiology and the North American Society of Pacing and Electrophysiology guidelines^9^. The HR was measured beat-to-beat during the procedures through a HR monitor (Polar RS800cx, Finland) with a sampling rate of 1 kHz. The IBIs that were recorded were transferred to the Polar Precision Performance Software (v. 3.0, Polar Electro, Finland) that allows visualization of signal stability. Five-minute intervals were chosen and saved in ".txt" file. Then, digital filtering was assumed complemented with manual filtering for the elimination of artifacts. Only series with greater than 95% of sinus beats were included^9^. Further details have been documented previously^27,28^.

The time domain index of HRV was studied via the root mean square of successive differences (RMSSD), the frequency domain index was evaluated by the high frequency spectral component (HF) of the power spectral density (0.15–0.4 Hz) in absolute units, the quantifiable Poincaré plot analysis was finalized via the SD1 index (standard deviation of the instantaneous beat-to-beat variability)^9^ and, symbolic analysis was achieved by the 0 V parameter^29^.

For data analysis we nominated a stable series of 256 IBIs for SD1, HF and 0 V parameters in addition to one with 60 IBIs for RMSSD.

The low frequency (LF) or the LF/HF ratio were excluded from the analysis as they have been demonstrated to be theoretically flawed and empirically unsupported to represent the sympatho-vagal balance^30,31^. The standard deviation of all normal IBIs (SDNN) and SD2 Poincaré plot index were excluded from examination as they are unable isolate the sympathetic and parasympathetic components^9^.

To compute the HRV indices, the Kubios HRV software package (Kubios HRV v.1.1, University of Kuopio, Finland) was enforced^32^.

#### Skin conductance

Skin conductance was evaluated via the eSense skin conductance system (Mindfield Biosystems, Inc., Berlin, Germany) on a Smartphone (Moto G4 Plus), which was previously validated to assess skin conductance^33^. Two finger electrodes were clipped to the middle and index fingers with Velcro straps. The electrodes were connected via the Smartphone through an audio connection input. Isotonic paste was added to the electrodes prior to attachment to the fingers to allow satisfactory contact with the skin. The eSense skin conductance system assimilated data at a 10 Hz sampling rate and the row data was transferred by email using .csv files.

### Interventions

#### Initial assessment

The experimental interventions were conducted 48–72 apart; allowing a sufficient recovery period for the subjects.

With the intention of controlling sources of bias, the study was completed between 17:00 and 22:00 to regulate circadian effects^34^ in a soundless room with humidity between 40 and 70% and temperature between 20 and 26 °C. The subjects were advised to refrain from drinking alcohol or performing exhaustive exercise 24 h before each protocol and to not consume caffeinated drinks eight hours prior to the experimental procedures. Subjects were told to dress in appropriate and comfortable clothing to allow the required physical effort and to eat only a light meal two hours prior to the procedures.

The descriptive characteristics of participants including age, SBP, DBP, mass, hip, abdominal and waist circumferences, waist-hip ratio, fat percentage, height, and body mass index (BMI) were standardized in order to facilitate reproducibility and physiological interpretation and control physiological variabilities. This standardization was founded on normal physiological parameters^26^.

#### Experimental protocols

As the initial step in the protocol, the subjects ingested 600 mg of avocado pulp or placebo (600 mg starch) in identical capsules 60 min prior to the exercise protocol. After ingesting the capsules, the subjects remained seated, continuing spontaneous breathing until the experimental procedures’ commencement.

Avocado pulp was prepared in Marilia, SP, Southeastern region of Brazil.

Then, the subjects began treadmill exercise with slope of 1% for the first 5 min to warm up (50–55% of maximal HR (HR_max_): 208 – 0.7 × age)^35^, after this, speed increased by increments of 0.5 km/h every minute until reaching submaximal HR, and then remained at this speed for 25 min (65–70% of HR_max_). Immediately after exercise, the subjects endured three minutes standing on the treadmill and were then seated for passive recovery for a further 57 min, totaling 60 min of recovery. During recovery from exercise the subjects remained seated silently with spontaneous breathing, they were unable to make any movements that would induce autonomic changes, could not sleep or ingest any type of food or drink.

HR, SBP and DBP were measured at the following times: Rest—55th to 60th minute of resting after capsule ingestion—and during recovery—1st, 2nd, 3rd, 5th, 7th, 10th, 20th, 30th, 40th, 50th and 60th minutes after exercise.

The RMSSD HRV index was calculated at the following times: Rest (M1: 55th to 60th minute of resting after capsule ingestion), exercise (M2: 15th to 20th minute of exercise) and during recovery: M3 (0 to 1st minute), M4 (1st to 2nd minute), M5 (2nd to 3rd minute), M6 (5th to 10th minute), M7 (15th to 20th minute), M8 (25th to 30th minute), M9 (35th to 40th minute), M10 (45th to 50th minute) and M11 (55th to 60th minute).

**Figure 2:**
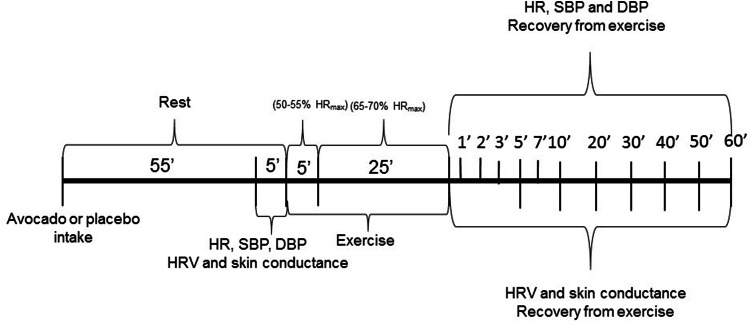
Study design.

Skin conductance, SD1, 0 V and HF HRV indices were taken at the following times: Rest (M1: 55th to 60th minute of resting after capsule ingestion), exercise (M2: 15th to 20th minute of exercise) and during recovery: M6 (5th to 10th minute), M7 (15th to 20th minute), M8 (25th to 30th minute), M9 (35th to 40th minute), M10 (45th to 50th minute) and M11 (55th to 60th minute)^7,36^ (Fig. 2).

All variables were measured with the subjects silent, with an empty bladder and spontaneous breathing.

### Sample size

The sample calculation was performed based on a pilot study. Online software was applied via the website http://www.lee.dante.br and computed the RMSSD index as a reference. A standard deviation of 12.8 ms was attained and the magnitude of the difference was 14.11 ms, with alpha risk of 5% and beta risk of 80%. The sample size projected was a minimum of 11 subjects per group.

### Randomization

The subjects and the researcher were blinded following assignment to interventions, throughout the whole experimental protocol. An investigator who did not participate in the study completed the random allocation sequence, enrolled participants and assigned participants to their suitable interventions.

### Statistical methods

The normality of the data was evaluated using the Shapiro–Wilk statistical test.

For decisions regarding the HRV indices, skin conductance and cardiorespiratory parameters between protocols and time period (rest vs. exercise vs. recovery), the data was primarily tested for sphericity violation via the Mauchly test. The Greenhouse–Geisser correction was required when the sphericity was violated. Comparisons of the values of variables between avocado vs. placebo protocols and their time period were achieved using two-way repeated measures ANOVA. For the analysis of points in time (rest vs. exercise vs. recovery) and the parametric distribution the repeated measurements ANOVA was implemented and after that the Bonferroni post-test. For non-parametric distributions the Friedman test was implemented followed by Dunn’s post-test. Significant differences were considered for p < 0.01, (or < 1%).

Effect sizes were calculated via Cohen's *d* to measure the magnitude of changes for significant differences. Large effect sizes were assumed for values greater than or equal to 0.9, medium effect sizes for values between 0.9 and 0.5 and small effect sizes for values between 0.5 and 0.25^37^.

Minitab software (Minitab, PA, USA) was used in addition to GraphPad InStat—v3.06, (GraphPad Software, Inc., San Diego California USA) and IBM SPSS Statistics—v22.0 (SPSS Inc. Chicago USA).

### Ethical approval and informed consent

All experimental protocols were evaluated and approved by the Research Ethics Committee in Research of UNESP/Marilia (Number 3.098.518). All participants signed a confidential informed letter of consent. All procedures were achieved in accordance with the 466/2012 resolution of the National Health Council of December 12th 2012.

## Results

### Sample profile

The sample characteristics with regards age, mass, height, BMI, waist circumference, abdominal circumference, hip circumference, waist-hip ratio, height, weight, fat percentage, estimated HR_max_, 50%, 55%, 65% and 70% of estimated HR_max_ of the 12 subjects are described in Table 1. Table 1 illustrates the homogeneity within the group for the presented variables.

**Table 1:** Mean values followed by their respective standard deviations, minimum and maximum of age, mass, height, BMI, waist circumference, abdominal circumference, hip circumference, waist-hip ratio, height, weight, fat percentage, estimated HR_max_, 50%, 55%, 65% and 70% of estimated HR_max_.

Variables	Values
Age (years)	20.75 ± 1.7 (19–25)
BMI (kg/m^2^)	21.16 ± 1.78 (18.9–24.1)
Height (m)	1.62 ± 0.07 (1.5–1.74)
Waist circumference (cm)	0.69 ± 0.04 (0.62–0.77)
Abdominal circumference (cm)	0.75 ± 0.05 (0.67–0.87)
Hip circumference (cm)	0.94 ± 0.06 (0.83–1.02)
Waist–hip ratio	0.73 ± 0.02 (0.69–0.77)
Mass (kg)	56.06 ± 7.47 (46–69.4)
Fat percentage (%)	31.95 ± 4.16 (25.9–39.7)
Estimated HR_max_ (bpm)	199.25 ± 1.71 (195–201)
50% HR_max_ (bpm)	99.37 ± 0.93 (97–100)
55% HR_max_ (bpm)	109.33 ± 0.98 (107–110)
65% HR_max_ (bpm)	129.25 ± 1.21 (126–130)
70% HR_max_ (bpm)	139.1 ± 1.33 (136–140)

*BMI* body mass index, *HR*_*max*_ maximum heart rate, *kg* kilogram, *m* meters, *bpm* beats per minute.

### Cardiorespiratory parameters

**Figure 3:**
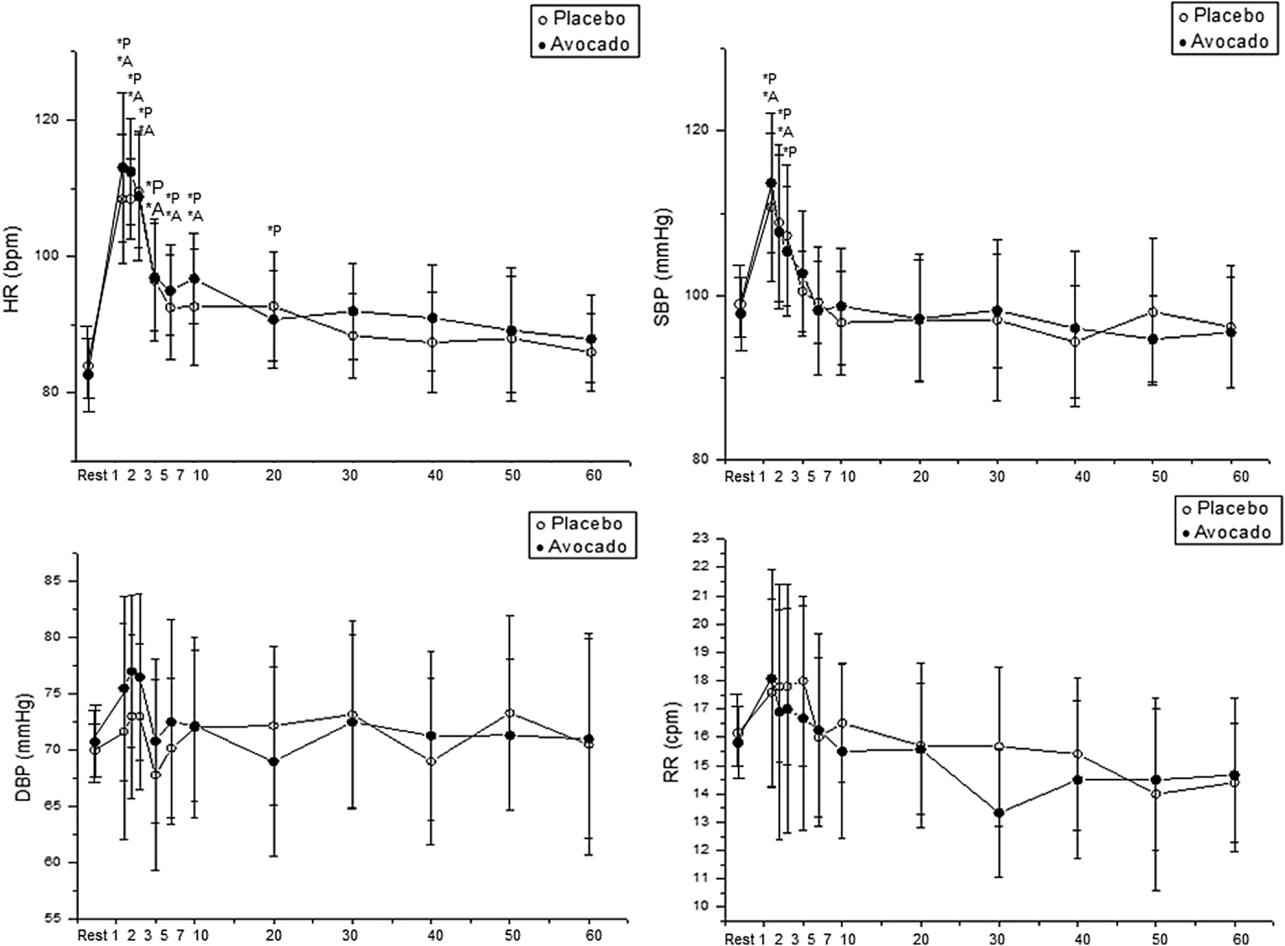
Mean values and respective standard deviations of HR, SBP, DBP and RR obtained at rest and during recovery from moderate aerobic exercise protocol. *P: values with significant differences in relation to rest (p < 0.01) for placebo protocol; *A: values with significant differences in relation to rest (p < 0.01) for avocado protocol. *SBP* systolic blood pressure, *DBP* diastolic blood pressure, *HR* heart rate, *RR* respiratory rate, *mmHg* millimeters of mercury, *bpm* beats per minute, *cpm* cycles per minute.

An effect of the time was stated for HR and SBP (p < 0.01). No effect was discovered between protocols for RR, SBP, DBP and HR. HR was higher during recovery from exercise compared to rest. In the avocado protocol, it was higher at 1 min, 2 min, 3 min, 5 min, 7 min and 10 min after exercise cessation. In the placebo protocol, HR increased 1 min, 2 min, 3 min, 5 min, 7 min, 10 min and 20 min following exercise cessation (Fig. 3).

For SBP, it was elevated 1 min and 2 min after exercise termination in the avocado protocol, but then it was higher 1 min, 2 min and 3 min after exercise in the placebo protocol. No significant changes were found in DBP and RR between rest and during recovery from exercise in either protocol (Fig. 3).

Effect size values for important changes via Cohen’s *d* are illustrated (Table 2).

**Table 2:** Effect size through Cohen’s *d* for SBP and HR.

	Rest vs. 1 min	Rest vs. 2 min	Rest vs. 3 min	Rest vs. 5 min		Rest vs. 7 min		Rest vs. 10 min		Rest vs. 20 min	
**Placebo**											
SBP	1.75	1.43	1.32	–		–		–		–	
HR	3.22	4.38	3.68	1.78		1.38		1.31		1.37	
**Avocado**											
SBP	1.94	1.13	–	–		–		–		–	
HR	3.19	3.95	2.97	1.71		1.59		1.87		–	

*SBP* systolic blood pressure, *HR* heart rate.

### Parasympathetic regulation of heart rhythm

**Figure 4:**
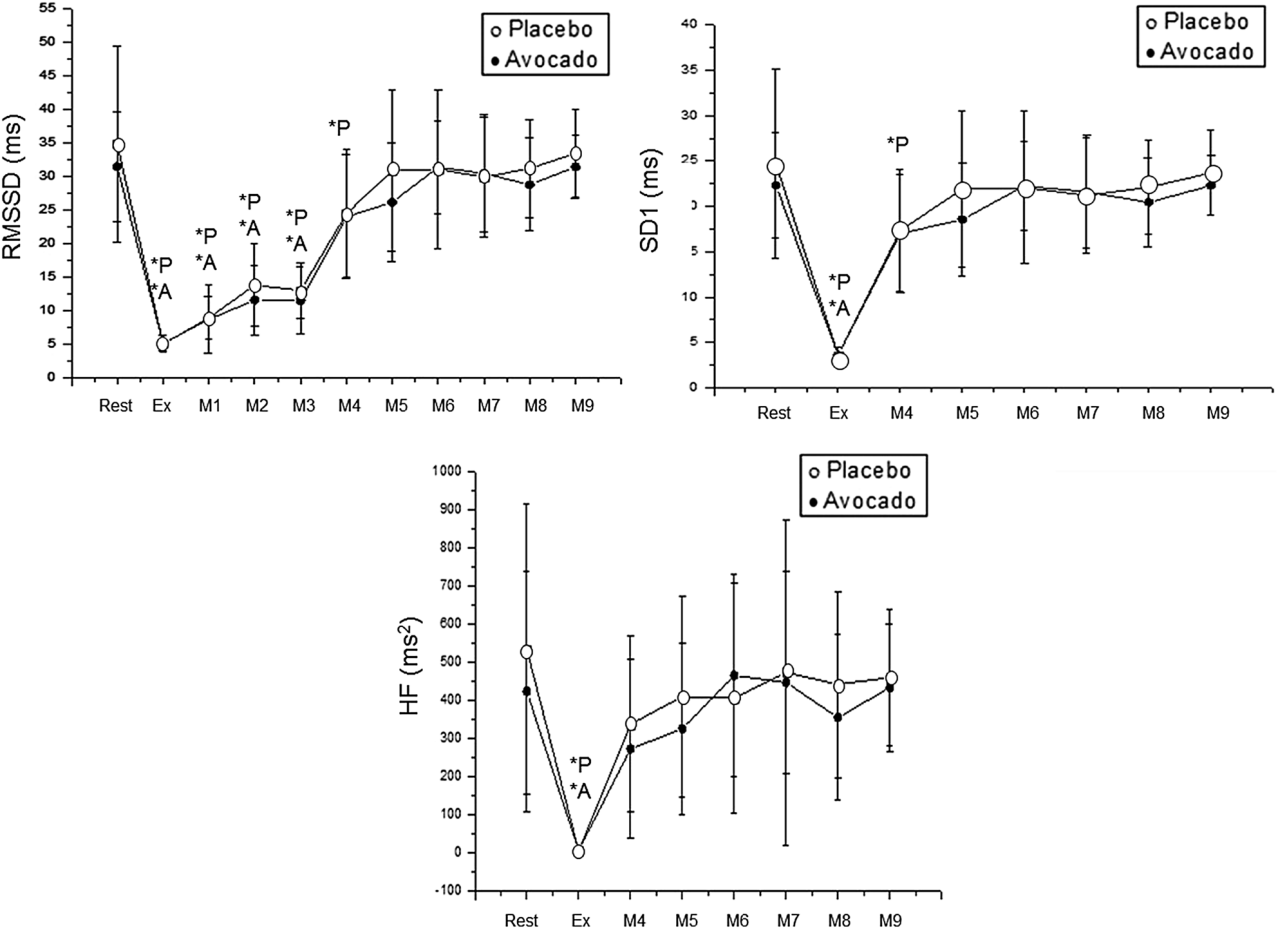
Mean values and respective standard deviations of RMSSD, SD1 and HF obtained at rest and during recovery from moderate aerobic exercise protocol. *P: values with significant differences in relation to rest (p < 0.01) for placebo protocol; *A: values with significant differences in relation to rest (p < 0.01) for avocado protocol. *RMSSD* square root of the square mean of the differences between adjacent normal IBI, *HF* high frequency, *SD1* standard deviation of instantaneous beat-to-beat variability, *ms* milliseconds, *ms*^*2*^ absolute units. Rest: 55th to 60th minute of resting after capsule ingestion; Ex: exercise—15th to 20th minute of exercise; M1: recovery from exercise—0 to 1st minute; M2: recovery from exercise—1st to 2nd minute; M3: recovery from exercise—2nd to 3rd minute; M4: recovery from exercise—5th to 10th minute; M5: recovery from exercise—15th to 20th minute; M6: recovery from exercise—25th to 30th minute; M7: recovery from exercise—35th to 40th minute; M8: recovery from exercise—45th to 50th minute; M9: recovery from exercise—55th to 60th minute).

Parasympathetic control of HR was studied via RMSSD, SD1 and HF indices. There was an effect of the moment on the parameters mentioned (p < 0.01), but no effect was observed between protocols. The RMSSD was reduced during exercise, 1 min, 2 min and 3 min after exercise in the avocado protocol. The same index was reduced during exercise and during the initial 10 min following exercise end in the placebo protocol (Fig. 4).

Regarding SD1, it was lessened during exercise in the avocado condition, while it was diminished during exercise and 5 to 10 min during recovery from exercise in the placebo protocol. The HF was reduced during exercise for both avocado and placebo protocols (Fig. 4).

Effect size values for significant differences through Cohen’s *d* are in Table 3.

**Table 3:** Effect size through Cohen’s *d* for HRV for autonomic variables.

	Rest vs. exercise	Rest vs. 1 min	Rest vs. 2 min	Rest vs. 3 min	Rest vs. 5–10 min	Rest vs. 15–20 min	Rest vs. 25–30 min	Rest vs. 35–40 min	Rest vs. 45–50 min
**Placebo**									
RMSSD	2.86	2.44	1.86	2.02	0.83	–	–	–	–
HF	1.95	–	–	–	–	–	–	–	–
SD1	2.58	2.43	–	–	–	–	–	–	–
0 V	1	–	–	–	–	–	–	–	–
Skin conductance	3.03	–	–	–	1.68	1.76	1.81	1.71	1.65
**Avocado**									
RMSSD	4.49	3.33	2.86	2.39	–	–	–	–	–
HF	1.54	–	–	–	–	–	–	–	–
SD1	4.49	–	–	–	–	–	–	–	–
0 V	–	–	–	–	–	–	–	–	–
Skin conductance	2.04	–	–	–	1.42	–	–	–	–

*RMSSD* square root of the square mean of the differences between adjacent normal IBI, *HF* high frequency, *SD1* standard deviation of instantaneous beat-to-beat variability, *HF* high frequency.

### Sympathetic autonomic nervous system

**Figure 5:**
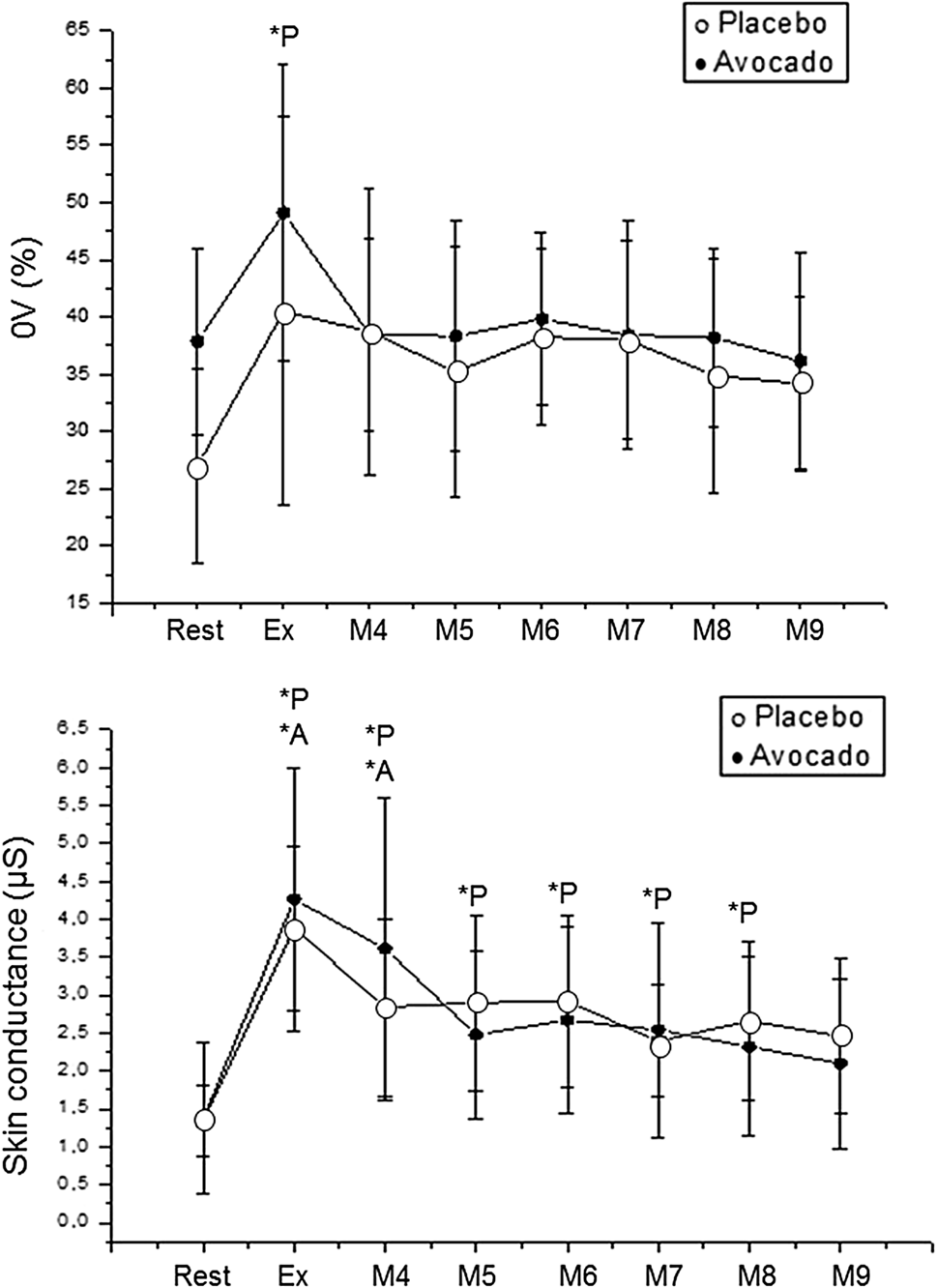
Mean values and respective standard deviations of 0 V parameter and skin conductance obtained at rest and during recovery from moderate aerobic exercise protocol. *P: values with significant differences in relation to rest (p < 0.01) for placebo protocol; *A: values with significant differences in relation to rest (p < 0.01) for avocado protocol. µS: microsiemens. Rest: 55th to 60th minute of resting after capsule ingestion; Ex: exercise—15th to 20th minute of exercise; M1: recovery from exercise—0 to 1st minute; M2: recovery from exercise—1st to 2nd minute; M3: recovery from exercise—2nd to 3rd minute; M4: recovery from exercise—5th to 10th minute; M5: recovery from exercise—15th to 20th minute; M6: recovery from exercise—25th to 30th minute; M7: : recovery from exercise—35th to 40th minute; M8: recovery from exercise—45th to 50th minute; M9: recovery from exercise—55th to 60th minute).

Sympathetic control was examined by means of 0 V symbolic parameter and skin conductance. An effect of the moment for 0 V and skin conductance was achieved in the placebo protocol, but an effect of the moment only on skin conductance was seen in the avocado protocol. Yet, no effect was observed between protocols. The 0 V symbolic parameter was increased during exercise in the placebo protocol, whereas no change was detected in the avocado protocol (Fig. 5).

Regarding skin conductance, it increased during exercise and 5–10 min following exercise termination in the avocado protocol. In the placebo protocol, it increased during exercise, 5–10 min, 15–20 min, 25–30 min, 35–40 min and 45–50 min during recovery from exercise (Fig. 5).

Table 3 illustrates effect size calculations for significant changes through Cohen’s *d*.

## Discussion

This study aimed to investigate the acute impact of avocado pulp ingestion on cardiorespiratory and autonomic recovery from exercise. Key results include: (1) avocado accelerated the SBP return to baseline after moderate effort; (2) recovery of HR after exercise was faster when subjects ingested avocado pulp; (3) re-entrance of HR parasympathetic control was quicker when under avocado administration and; (4) HR sympathetic regulation and skin conductance recovery after moderate exercise were improved in the avocado pulp condition.

Evaluation of cardiovascular and autonomic recovery after exercise is an important measure to detect physiological difficulties directly after effort, including increased risk of cardiovascular events and sudden death. This technique provides evidence hidden during resting conditions^5,6^. Thus, this investigation intended to concentrate on non-pharmacological interventions before exercise to identify alternatives to lessen cardiovascular complications after exertion^38–40^. Hence, to authenticate conceivable components to be enforced by subjects succumbed to an exercise training plan.

Recent studies have intended to evaluate the cardioprotective effect of avocado^19,41,42^. The aforesaid references demonstrated the valuable influences of avocado fruit, avocado oil or avocado extract. This study has provided primary evidence that acute avocado pulp supplementation induced a positive effect for cardiovascular and autonomic recovery after exercise.

Based on these results, avocado pulp improved SBP and HR recovery from moderate effort. When the subjects were ingested avocado pulp, SBP recovered after 2 min following exercise and HR returned after 10 min after exercise termination, while in the placebo protocol, SBP recovered after 3 min following exercise cessation and HR recovered after 20 min of recovery. The abovementioned findings are in accordance with Dzeufiet et al.^41^, who studied animals treated with aqueous avocado extract (50, 100 and 150 mg/kg) for five weeks. Their results confirmed that aqueous avocado extract dose-dependently reduced HR and blood pressure of ethanol-sucrose induced hypertensive rats. The current data supports the use of nitric oxide and the vasodilator properties of avocado discovered by Dzeufiet et al.^41^. Similarly, this study provides introductory results indicating its acute effect on blood pressure in response to exercise.

In agreement with the current data, Márquez-Ramírez et al.^43^, compared the chronic effects of avocado oil and losartan on blood pressure in hypertensive rats induced with Nω-nitro-l-arginine methyl ester (L-NAME). Animals were administered avocado oil for 45 days. The study demonstrated beneficial effects of chronic avocado oil on cardiovascular physiology, including lowered systemic blood pressure, reduced renal reactive oxygen species, improved renal endothelium-dependent vasodilation and kidney mitochondrial function, suggesting that avocado positively interacts with the angiotensin system in regulating blood pressure, while the current study evaluated the acute effects of avocado ingestion.

Another mechanism involved in the influence of avocado on blood pressure is attributed to its antioxidant properties^44^. Earlier, thiobarbituric acid reactive species analysis, hydrophilic and lipophilic oxygen radical absorbance capacity values indicated an important acute antioxidant characteristic of acetogenins in avocado, asserting it as an innovative lipophilic antioxidant present in avocado pulp^44^. Thus, biochemical and oxidative status improved by chronic avocado treatment was linked with decreased blood pressure and HR in ethanol-sucrose induced hypertensive rats^41^, supporting the existing results concerning the acute influence of avocado.

Yet, it was unclear if there was any impact of avocado pulp on the health of subjects acquiesced to a stress test, such as an acute exercise bout. The existing data proposes that acute avocado pulp consumption has an advantageous effect on blood pressure fluctuations induced by exercise in healthy females. One more vital point to be addressed is that this study emphasizes the acute effects of avocado pulp, yet its mechanism has not been well elucidated until now.

Consistent with the existing data, beneficial changes in blood pressure and HR recovery from exercise triggered by avocado pulp is to some extent explained due to the effects avocado has on autonomic function. This is because HRV and skin conductance offered a faster return to baseline levels after moderate exercise when avocado pulp was ingested. It was shown that when the subjects administered avocado pulp before exercise, RMSSD recovered after 3 min following exercise, and SD1 returned after 5–10 min after exercise. However, when they ingested placebo capsules, RMSSD and SD1 recovered after 10 min of recovery from moderate effort, indicating that HR autonomic regulation was raised by the avocado pulp.

HRV analysis demonstrated that vagal recovery after exercise was faster owing to avocado pulp intake, and this explanation is strengthened by quicker recovery of HR in the avocado protocol. In contrast, after a thorough search in the PubMed/Medline, Scopus and Web of Science databases, to date, no study has evaluated the effect of avocado on autonomic nervous system. In this context, the quicker vagal recovery from exercise in the subjects acutely treated with avocado may be somewhat explained by HR changes induced by avocado found by Dzeufiet et al.^41^.

Likewise, skin conductance recovered before 5 min after exercise in the avocado protocol, whereas it returned to baseline after 45–50 min after exercise in the placebo protocol. This study stated that the 0 V symbolic parameter was increased during exercise in the placebo protocol, even though there was no significant adjustment observed when the participants were administrated avocado. These results advocate that avocado pulp has useful properties on sympathetic changes during moderate effort.

Similarly, the antioxidant profile of avocado^44^ might be involved in the quicker autonomic recovery after exercise. During physical effort, blood pressure and HR modulation are transformed, baroreflex sensitivity is reduced at brainstem level in response to metaboreflex activation. Elevated cellular metabolism promotes metabolic accumulation and activates metaboreceptors. Therefore, the stimulation of non-myelinated afferent fibers elevates sympathetic activity, triggering increased HR, cardiac output, blood pressure and vasoconstriction in non-active muscles^45^. After exercise, progressive removal of metabolites during recovery from exercise decreases metaboreflex activation, restoring baroreflex activity^3^. It was theorized that avocado hastens metabolic removal because of its antioxidant effects^44^.

Of late, Harms et al.^46^ evaluated the role of oxidative stress in the exercise pressor reflex. The study evaluated HR and pressor responses to static contraction of the triceps surae muscles in Sprague–Dawley rats during infusion of Tiron (a likely antioxidant) in those rats with simulated peripheral artery disease. The study uncovered that Tiron treatment reduced the exercise pressor reflex, signifying that antioxidant interventions are able to weaken blood pressure and HR responses to exercise in rats in that condition. Thus, it is proposed that the antioxidant effect of avocado may support a quickening in autonomic and cardiovascular recovery after exercise.

The hypothesis founded on the involvement of nitric oxide in the improvement of autonomic recovery from exercise triggered by avocado may be rejected. Though Dzeufiet et al.^41^ revealed that aqueous avocado extract improved nitrites content in ethanol-sucrose induced hypertensive rats, Senador et al.^47^ discovered that endothelial nitric oxide does not have a significant impact in systemic vasodilation during muscle metaboreflex activation in canines.

The current study highlights some concepts. Firstly, this study has recruited 12 healthy, physically active, college educated, female subjects to avoid influence of sexual hormones, improve reproducibility and the physiological clarification. Although, while a sample size calculation approved this sample, these results cannot be generalized to diverse genders, age, fitness status, or other medical conditions such as cardiovascular disease. This study did not isolate any constituent of avocado pulp in order to make the situation similar to the real life setting, given that general population have easier access to the entire avocado pulp rather than its specific components. In this sense, no conclusive argument can be made as to what exact bioactive component(s) of avocado pulp was responsible for our observations.

These results provide preliminary data supportive of the use of avocado pulp before exercise. Given that avocado supplement enhanced cardiovascular and autonomic recovery after moderate effort, this study advocates that this intervention may be considered for reducing cardiovascular complications immediately after exercise. This outcome is promising in particular, for individuals considering to engage in physical activity under medical supervision. This study proposes that each person should consult with a clinician to check any contraindications.

## Conclusion

The current results indicate that avocado pulp prior to aerobic exercise improves a number of cardiovascular and autonomic variables during recovery in healthy females. This outcome supports the advantageous effects of avocado pulp prior to exertion, suggesting the beneficial use of avocado before exercise.

## References

[1] Colombari E (2001). Role of the medulla oblongata in hypertension. Hypertension.

[2] Valenti VE (2010). ATZ (3-amino-1,2,4-triazole) injected into the fourth cerebral ventricle influences the Bezold–Jarisch reflex in conscious rats. Clinics.

[3] Fadel PJ (2015). Reflex control of the circulation during exercise. Scand. J. Med. Sci. Sports.

[4] Behrens M (2015). Caffeine-induced increase in voluntary activation and strength of the quadriceps muscle during isometric, concentric and eccentric contractions. Sci. Rep..

[5] Pecanha T, Silva-Junior ND, Forjaz CL (2014). Heart rate recovery: autonomic determinants, methods of assessment and association with mortality and cardiovascular diseases. Clin. Physiol. Funct. Imaging.

[6] Cole CR, Blackstone EH, Pashkow FJ, Snader CE, Lauer MS (1999). Heart-rate recovery immediately after exercise as a predictor of mortality. N. Engl. J. Med..

[7] Nishime EO, Cole CR, Blackstone EH, Pashkow FJ, Lauer MS (2000). Heart rate recovery and treadmill exercise score as predictors of mortality in patients referred for exercise ECG. JAMA.

[8] Jouven X (2005). Heart-rate profile during exercise as a predictor of sudden death. N. Engl. J. Med..

[9] Fontes A, de Oliveira LS, Vanderlei FM, Garner DM, Valenti VE (2018). Waist–stature ratio and its relationship with autonomic recovery from aerobic exercise in healthy men. Sci. Rep..

[10] Michael S, Graham KS, Davis GMO (2017). Cardiac autonomic responses during exercise and post-exercise recovery using heart rate variability and systolic time intervals—a review. Front. Physiol..

[11] Camm AJ (1996). Heart rate variability: standards of measurement, physiological interpretation and clinical use. Task Force of the European Society of Cardiology and the North American Society of Pacing and Electrophysiology. Circulation.

[12] Rossi DM, Valenti VE, Navega MT (2011). Exercise training attenuates acute hyperalgesia in streptozotocin-induced diabetic female rats. Clinics (Sao Paulo).

[13] Ferreira Filho C (2010). Anti-hypertensive drugs have different effects on ventricular hypertrophy regression. Clinics.

[14] Asgari S, Khaloo P, Khalili D, Azizi F, Hadaegh F (2019). Status of hypertension in Tehran: potential impact of the ACC/AHA 2017 and JNC7 guidelines, 2012–2015. Sci. Rep..

[15] Rang HP, Dale MM (2016). Rang and Dale's Pharmacology.

[16] Null G, Pennesi L, Feldman M (2017). Nutrition and lifestyle intervention on mood and neurological disorders. J. Evid. Based Complement. Altern. Med..

[17] Segovia FJ, Hidalgo GI, Villasante J, Ramis X, Almajano MP (2018). Avocado seed: a comparative study of antioxidant content and capacity in protecting oil models from oxidation. Molecules.

[18] Zhao CN (2017). Fruits for prevention and treatment of cardiovascular diseases. Nutrients.

[19] Carvajal-Zarrabal O (2014). Avocado oil supplementation modifies cardiovascular risk profile markers in a rat model of sucrose-induced metabolic changes. Dis. Markers.

[20] Rodriguez-Sanchez DG (2015). Isolation and chemical identification of lipid derivatives from avocado (*Persea**americana*) pulp with antiplatelet and antithrombotic activities. Food Funct..

[21] Park E, Edirisinghe I, Burton-Freeman B (2018). Avocado fruit on postprandial markers of cardio-metabolic risk: a randomized controlled dose response trial in overweight and obese men and women. Nutrients.

[22] Ojewole JA, Kamadyaapa DR, Gondwe MM, Moodley K, Musabayane CT (2007). Cardiovascular effects of *Persea**americana* Mill (Lauraceae) (avocado) aqueous leaf extract in experimental animals. Cardiovasc. J. Afr..

[23] Wang L, Bordi PL, Fleming JA, Hill AM, Kris-Etherton PM (2015). Effect of a moderate fat diet with and without avocados on lipoprotein particle number, size and subclasses in overweight and obese adults: a randomized, controlled trial. J. Am. Heart Assoc..

[24] Cuschieri S (2019). The CONSORT statement. Saudi J. Anaesth..

[25] Bai X, Li J, Zhou L, Li X (2009). Influence of the menstrual cycle on nonlinear properties of heart rate variability in young women. Am. J. Physiol. Heart Circ. Physiol..

[26] Rzewnicki R, Vanden Auweele Y, De Bourdeaudhuij I (2003). Addressing overreporting on the International Physical Activity Questionnaire (IPAQ) telephone survey with a population sample. Public Health Nutr..

[27] Hallal PC (2010). Validity and reliability of the telephone-administered international physical activity questionnaire in Brazil. J. Phys. Act. Health.

[28] 26Lohman, T. G., Roche, A. F. & Martorelli, R. *Antropometric Standardization Reference Manual* (1988).

[29] Vidigal GA (2016). Slow breathing influences cardiac autonomic responses to postural maneuver: slow breathing and HRV. Complement. Ther. Clin. Pract..

[30] Ferreira LL (2015). Response of cardiac autonomic modulation after a single exposure to musical auditory stimulation. Noise Health.

[31] Porta, A. *et al.* Entropy, entropy rate, and pattern classification as tools to typify complexity in short heart period variability series. *IEEE Trans. Biomed. Eng.* **48**, 1282–1291. https://doi.org/10.1109/10.959324 (2001). 10.1109/10.959324 11686627

[32] Billman GE (2013). The LF/HF ratio does not accurately measure cardiac sympatho-vagal balance. Front. Physiol..

[33] Heathers J (2012). Sympathovagal balance from heart rate variability: an obituary. Exp. Physiol..

[34] Niskanen JP, Tarvainen MP, Ranta-Aho PO, Karjalainen PA (2004). Software for advanced HRV analysis. Comput. Methods Programs Biomed..

[35] Hinrichs R (2017). Mobile assessment of heightened skin conductance in posttraumatic stress disorder. Depress. Anxiety.

[36] Singh RB (2003). Circadian heart rate and blood pressure variability considered for research and patient care. Int J Cardiol.

[37] Machado FA, Denadai BS (2011). Validade das equações preditivas da frequência cardíaca máxima para crianças e adolescentes. Arq. Bras. Cardiol..

[38] Gomes RL (2018). The effects of musical auditory stimulation on cardiorespiratory variables after aerobic exercise. Sci. Sports.

[39] Quintana DS (2017). Statistical considerations for reporting and planning heart rate variability case–control studies. Psychophysiology.

[40] Moreno IL (2013). Effects of an isotonic beverage on autonomic regulation during and after exercise. J. Int. Soc. Sports Nutr..

[41] Gonzaga LA, Vanderlei LCM, Gomes RL, Garner DM, Valenti VE (2019). Involvement of cardiorespiratory capacity on the acute effects of caffeine on autonomic recovery. Medicina (Kaunas).

[42] Gonzaga LA, Vanderlei LCM, Gomes RL, Valenti VE (2017). Caffeine affects autonomic control of heart rate and blood pressure recovery after aerobic exercise in young adults: a crossover study. Sci. Rep..

[43] Dzeufiet PD (2014). Antihypertensive potential of the aqueous extract which combine leaf of *Persea**americana* Mill. (Lauraceae), stems and leaf of *Cymbopogon**citratus* (D.C) Stapf. (Poaceae), fruits of *Citrus**medical* L. (Rutaceae) as well as honey in ethanol and sucrose experimental model. BMC Complement. Altern. Med..

[44] Mahmassani HA, Avendano EE, Raman G, Johnson EJ (2018). Avocado consumption and risk factors for heart disease: a systematic review and meta-analysis. Am. J. Clin. Nutr..

[45] Marquez-Ramirez CA (2018). Comparative effects of avocado oil and losartan on blood pressure, renal vascular function, and mitochondrial oxidative stress in hypertensive rats. Nutrition.

[46] Rodriguez-Sanchez D (2013). Activity-guided identification of acetogenins as novel lipophilic antioxidants present in avocado pulp (*Persea**americana*). J. Chromatogr. B Analyt. Technol. Biomed. Life Sci..

[47] Belli JFC, Bacal F, Bocchi EA, Guimarães GV (2011). Comportamento do ergorreflexo na insuficiência cardíaca. Arq. Bras. Cardiol..

[48] Harms JE, Kuczmarski JM, Kim JS, Thomas GD, Kaufman MP (2017). The role played by oxidative stress in evoking the exercise pressor reflex in health and simulated peripheral artery disease. J. Physiol..

[49] Senador D (2017). Role of endothelial nitric oxide in control of peripheral vascular conductance during muscle metaboreflex activation. Am. J. Physiol. Regul. Integr. Comp. Physiol..

